# Whole-exome sequencing reveals rare genetic variations in ovarian granulosa cell tumor

**DOI:** 10.17305/bjbms.2021.6789

**Published:** 2021-11-30

**Authors:** Seungyeon Kim, Songmi Kim, Seyoung Mun, Yongsik Kwak, Kwang-Sun Suh, Song-Yi Choi, Kyudong Han

**Affiliations:** 1 Department of Microbiology, College of Science and Technology, Dankook University, Cheonan, Republic of Korea; 2 Center for Bio-Medical Engineering Core Facility, Dankook University, Cheonan, Republic of Korea; 3 Department of Nanobiomedical Science, Dankook University, Cheonan, Republic of Korea; 4 Department of Pathology, School of Medicine, Chungnam National University, Daejeon, Republic of Korea

**Keywords:** Whole-exome sequencing, ovarian granulosa cell tumor, single-nucleotide polymorphism, indels, ovarian cancer

## Abstract

Ovarian granulosa cell tumor (OGCT) is a rare ovarian tumor that accounts for about 2-5% of all ovarian tumors. Despite the low grade of ovarian tumors, high and late recurrences are common in OGCT patients. Even though this tumor usually occurs in adult women with high estrogen levels, the cause of OGCT is still unknown. To screen genetic variants associated with OGCT, we collected normal and matched-tumor formalin-fixed paraffin-embedded from 11 OGCT patients and performed whole-exome sequencing using Illumina NovaSeq 6000. A total of 1,067,219 single nucleotide polymorphisms (SNPs) and 162,155 insertions/deletions (indels) were identified from 11 pairs of samples. Of these, we identified 44 tumor-specific SNPs in 22 genes and four tumor-specific indels in one gene that were common to 11 patients. We used three cancer databases (TCGA, COSMIC, and ICGC) to investigate genes associated with ovarian cancers. Nine genes (*SEC22B*, *FEZ2*, *ANKRD36B*, *GYPA*, *MUC3A*, *PRSS3*, *NUTM2A*, *OR8U1*, and *KRTAP10-6*) associated with ovarian cancers were found in all three databases. In addition, we identified seven rare variants with MAF ≤ 0.05 in two genes (*PRSS3* and *MUC3A*). Of seven rare variants, five variants in *MUC3A* are potentially pathogenic. Furthermore, we conducted gene enrichment analysis of tumor-specific 417 genes in SNPs and 106 genes in indels using cytoscape and metascape. In GO analysis, these genes were highly enriched in “selective autophagy,” and “regulation of anoikis.” Taken together, we suggest that *MUC3A* is implicated in OGCT development, and *MUC3A* could be used as a potential biomarker for OGCT diagnosis.

## INTRODUCTION

Ovarian granulosa cell tumor (OGCT) is a rare sex cord-stromal tumor that Rokitansky first described in 1855 [1]. It accounts for only 2-5% of all ovarian tumors and is estimated to occur in 0.6-1.0/100,000 women annually worldwide [2,3]. The incidence of OGCT is highest in postmenopausal women, especially between the ages of 50 and 55, and juvenile GCT occurs in <5% of pre-pubescent girls and women younger than 30 years of age [4,5]. The symptoms of OGCT are vaginal bleeding, pain abdomen, abdominal distension, menstrual abnormalities, or amenorrhea [2].

About 70-80% of OGCTs are diagnosed at stage I disease, the 10-year survival rate in stage I is 84-95%, decreases to 50-65% in stage II, and 17-33% in stages III and IV [6]. Although OGCT has a low grade, high and late recurrences are common in patients with OGCT [7]. Recurrence occurs in about 50% of patients, and it is known that 50-80% of patients die from recurrence [8]. When recurrence occurs, the prognosis for the patient is poor, and conventional chemotherapy is not effective for recurrence [9]. Although this tumor is usually known to occur in adult women with high estrogen levels, the cause of OGCT is still unclear [10].

To date, several types of research have been conducted to elucidate the pathogenesis and treatment of OGCT. Shah *et al*. reported that more than 95% of OGCT patients were found to have a *FOXL2* c.402C>G point mutation (C134W), which is a crucial transcription factor that regulates ovarian development and function [11-13]. Interestingly, WGS revealed that the *FOXL2* (c.402C>G) mutation is specific for OGCT but not commonly found in other cancers [12]. *FOXL2* regulates the crucial signaling pathways in the ovary, such as TGF-b/BMP signaling, MAP-kinase signaling, steroid signaling, PI3K/Akt signaling, involved in cell proliferation and apoptosis [14]. A recent study by Alexiadis et al. reported the high frequency of the *TERT* g. -124C>T mutation in the recurrent adult GCT [15]. *TERT* encodes the catalytic subunit of telomerase involved in oncogenesis. The mutation of the *TERT* promoter is a biomarker for the prognosis of various cancers, including hepatocellular carcinoma, chondrosarcoma, and primary glioblastoma [16-18]. Despite efforts to understand the development and recurrence of OGCT, the pathogenesis is still insufficient. Here, we aimed to detect genetic variants involved in OGCT development in normal and matched-tumor tissues from 11 OGCT patients by whole-exome sequencing (WES).

## MATERIALS AND METHODS

### Patient

A total of 11 OGCT patients from the Chungnam university hospital were included in this study. The age of patients was 27-78-years-old. Patients were diagnosed at various times by individuals from 2011 to 2017. We collected normal and matched-tumor formalin-fixed paraffin-embedded (FFPE) from 11 OGCT patients. The tumor with an average size of about 10 cm (range 3.3-22.5 cm) was collected from each of the 11 patients (Table 1). According to the manufacturer’s instructions, DNAs were extracted from twenty-two FFPE samples from 11 patients using the Maxwell 16 FFPE plus LEV DNA purification kit (Promega, USA). The paired normal tissue was the contralateral ovarian tissue from each patient. The pathologist then made a microscopic diagnosis of normal and tumor tissue using hematoxylin and eosin-stained biopsy slides.

**TABLE 1:**
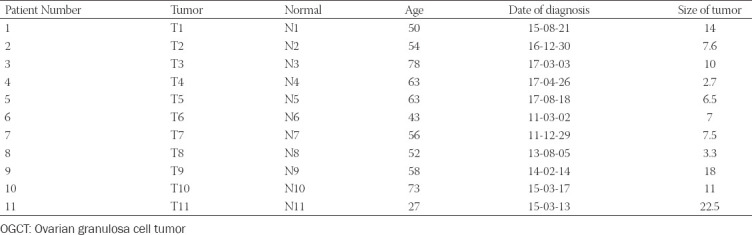
Clinical characteristics of patients with OGCT

### WES and variant calling

Preparation for capturing libraries with an Agilent SureSelect Target Enrichment Kit (Agilent, USA) followed the manufacture’s protocols. The libraries were sequenced with an Illumina NovaSeq 6000 with a 2 × 150 bp paired-end read. After that, sequencing reads were aligned to the human reference genome using the Burrows-Wheeler Alignment tool (BWA 0.7.12) with -M parameters. Picard (picard-tools-1.130) was used to remove PCR duplicates, and the Genome Analysis Tool kit (GATKv3.4.0) was performed for variant calling with -T and -knownSites parameters. Here, we only used the variants more than 30 depths in coverage. Functional annotation was conducted using SnpEff (SnpEff_v4.1g) with default settings.

### Cancer databases

We used The Catalogue Of Somatic Mutations In Cancer (COSMIC), International Cancer Genome Consortium (ICGC), and The Cancer Genome Atlas (TCGA) to find ovarian cancer-related genes. Three databases contain mutational signatures in the cancer genome.

### Gene set enrichment analysis

We performed a gene ontology (GO) enrichment analysis of the variants with tumor-specific genes to investigate the biological relevance of the candidate genes using Metascape software ( https://metascape.org/gp/index.html). The significant gene sets are classified into three classes: biological process (BP), cellular component (CC), and molecular function (MF). In addition, to determine their biological functions related to cancer and associated pathway, we performed molecular and genetic interaction networks analysis using Cytoscape software (cytoscape_v3.7.0 and ClueGO_v.2.5.7). We used the Benjamini and Hochberg (BH) adjustment to correct the *p*-value in ClueGO.

### Pathogenic variants analysis

We used three databases to analyze the Exome Aggregation Consortium (ExAC) (n = 60,706, http://exac.broadinstitute. org/), 1000 Genomes project phase 3 database (1000 G; n = 2,504, http://www.internationalgenome.org/), and National Heart, Lung, and Blood Institute (NHLBI) (n = 6,503, http://evs.gs.washington.edu/EVS/) to investigate the rare variants in the general population. We collected nonsynonymous variants with MAF ≤ 0.05, shared by 11 patients. In addition, we performed a pathogenic analysis of the variants to predict protein functional effects using Sorting Intolerant From Tolerant (SIFT) (http://sift.jcvi.org) and PolyPhen-2 (http://genetics.bwh.harvard.edu/pph2/) tools.

### Ethics statement

The study protocol was approved by the Institutional Review Board of Chungnam National University Hospital and complied with the tenets of the Declaration of Helsinki (2016-12-056).

## RESULTS

### Subjects and WES

We recruited 11 patients from the Chungnam university hospital. The mean age was 56 (range 27-78) years. The size of the tumor is an average of 10.00 cm (Table 1). We obtained 22 fresh-frozen samples, including 11 normal and matched- tumor FFPE from OGCT patients for WES. On Illumina NovaSeq 6000 platform with 150 bp paired-end reads, WES data were generated with an average of 128 Gb sequences. The post-alignment average read depth of the WES was 258X and 169.7X in tumor and normal samples, respectively. Sequencing quality for Q30 value was 91.5% and 92.8% in tumor and normal tissues, respectively (Table S1). Complete supplementary data is available at https://www.bjbms.org/ojs/index.php/bjbms/article/view/6789/2433

### Identification of OGCT related variants

**FIGURE 1:**
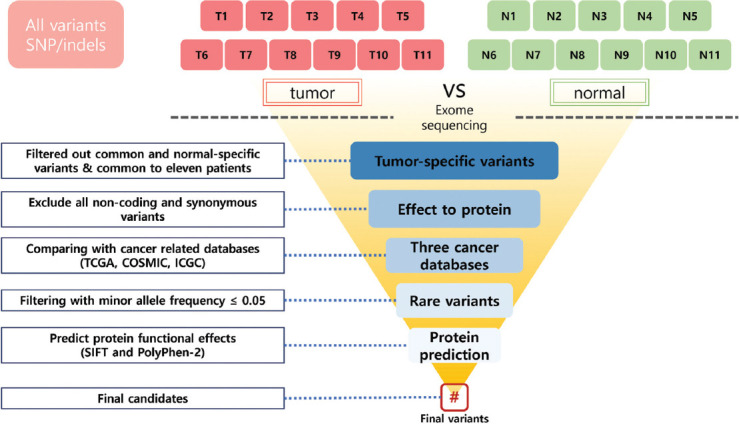
Comparison criteria for OGCT rare variant with 11 patients. The normal and cancer tissue isolated from 11 OGCT patients were involved in identification of rare mutation using whole-exome sequencing. Green and red boxes indicate normal and matched-tumor samples, respectively. The scope was narrowed step by step, and the critical nonsynonymous variants closely related to OGCT were finally identified by evaluating the influence of phenotype. OGCT: Ovarian granulosa cell tumor.

A total of 1,067,219 single nucleotide polymorphisms (SNPs) and 162,155 insertions/deletions (indels) were identified from WES data of 22 samples. To identify OGCT related variants, we collected 29,998 SNPs and 3,437 indels, shared by 11 patients (Table 2; Table S2). In the variant calling step, we selected only the variants with at least 30 depth coverage to eliminate possible errors in library preparation and sequencing data production and to determine the substantial variants in OGCT. As a result, variants with 31.7 minor depth and 417 average depth were selected. To identify OGCT related variants, we identified 7,957 SNPs and 234 indels in the exonic region (Table 2). Of these, we identified 4110 nonsynonymous variants, including missense, nonsense, and unknown variants, and 137 frameshift indels, including nonsense and unknown variants that could affect protein functions (Figure 1; Table 3). To analyze the variants associated with OGCT development and recurrence, we focused on 44 tumor-specific nonsynonymous variants in 22 genes (*PABPC3, ZNF595, MUC3A, OR2T4, SEC22B, PRSS3, NBPF14, NBPF19, NBPF26, NBPF9, FEZ2, ANKRD36C, ANKRD36B, GYPA, PRIM2, VWDE, CFTR, NUTM2A, MUC6, OR8U1;OR8U8, GOLGA6L3, TPSD1, NPIPB15, SHC2*, and *KRTAP10-6*) and four tumor-specific frameshift indels in *ZNF595* (Table 3). *PABPC3* encodes a protein that binds to the poly (A) tail and regulates mRNA stability. Previous studies reported that WES reveals high-risk frameshift mutations and somatic mutations for *PABPC3* in breast cancer patients [19,20] and malignant ovarian germ cell tumors, respectively [21]. *ZNF595* encodes a protein belonging to the C_2_H_2_ zinc finger protein family that is involved in transcriptional regulation. Recent studies revealed that zinc finger protein families are closely related to various cancer and tumor types, such as tumorigenesis or tumor suppressor genes [22]. For example, overexpression of *ZNF304* transcriptionally regulates β1 integrin, resulting in metastasis of ovarian cancer [23]. β1 integrin, a member of the integrin family, is involved in cell adhesion and recognition in immune response, tissue repair, and tumor cell metastasis. In addition, *ZKSCAN3* (*ZFN306*) also contributes to tumor metastasis in colorectal cancer cells, prostate cancers, and hepatocellular carcinoma [24-26]. For a 5-year survival rate, *ZKSCAN3* is used for the potential prognostic marker of hepatocellular carcinoma patients. *ZKSCAN3* increases the expression of *ITGB4* (integrin b4) binding to its promoter, resulting in promoting migration, invasion, and EMP progress. *ITGB4* activates the AKT signaling pathway involved in cell proliferation [26].

**TABLE 2:**
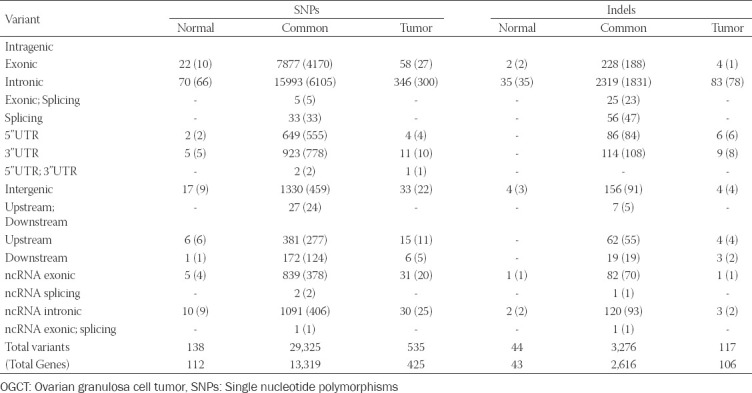
The number of candidate variants in OGCT patients

**TABLE 3:**
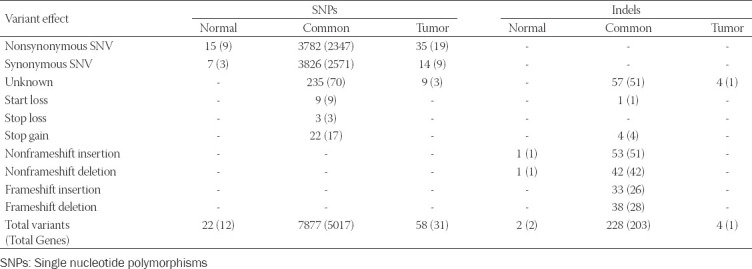
Effects of variants in the exonic regions

**FIGURE 2:**
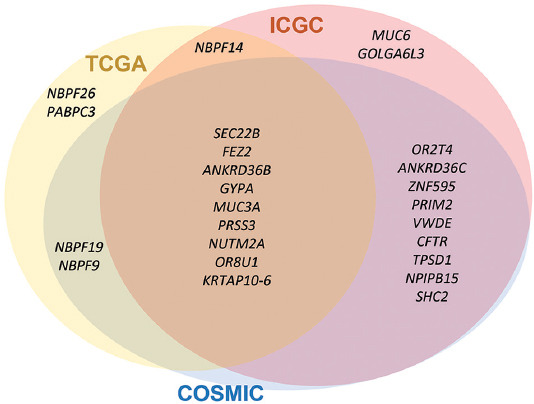
Venn diagram of the three cancer-related databases (ICGC, TCGA, and COSMIC). The Venn diagram shows the genes harboring tumor-specific nonsynonymous variants matched with each database, including TCGA (yellow), ICGC (pink), and COSMIC (blue) cancer genome projects. Nine were included in the common set as commonly identified genes in all databases. COSMIC: The Catalogue Of Somatic Mutations In Cancer, ICGC: International Cancer Genome Consortium, TCGA: The Cancer Genome Atlas.

Furthermore, we investigated 22 genes for whether the genes were related to OGCT using three cancer-related databases (TCGA, ICGC, and COSMIC) that contain cancer-associated genes across all cancer types. We found nine genes (*SEC22B*, *FEZ2*, *ANKRD36B*, *GYPA*, *MUC3A*, *PRSS3*, *NUTM2A*, *OR8U1*, and *KRTAP10-6)* in all three databases, which were highly associated with ovarian cancer (Figure 2). *FEZ2* is a family of FEZ proteins involved in axonal growth in *Caenorhabditis elegans*. FEZ proteins are involved in neuronal development, neurological disorders, viral infection, and autophagy. *FEZ1* is a tumor suppressor gene and is implicated in ovarian carcinogenesis. *FEZ1* was evaluated as a prognostic and diagnostic marker for ovarian neoplasia [27]. *NUTM2A* (NUT family member 2A), also known as *FAM22A*, reported that *YWHAE*-*NUTM2A* fusion transcript is associated with aggressive endometrial stromal sarcomas [28]. *SEC22B*, a member of the *SEC22* family of vesicle-trafficking proteins, is involved in the membrane fusion of vesicle trafficking between the endoplasmic reticulum and Golgi apparatus, secretory autophagy, and antigen cross-presentation [29]. Several studies have reported that *SEC22B* is highly related to tumorigenesis that the mutations in *SEC22B* were found in various cancers. Interestingly, the fusion of *SEC22B*-NOTCH2 activates the NOTCH pathway to the proliferation and survival of tumor cells in aggressive breast cancers and mantle cell lymphoma [30-32].

### Enrichment and pathway analysis

**FIGURE 3:**
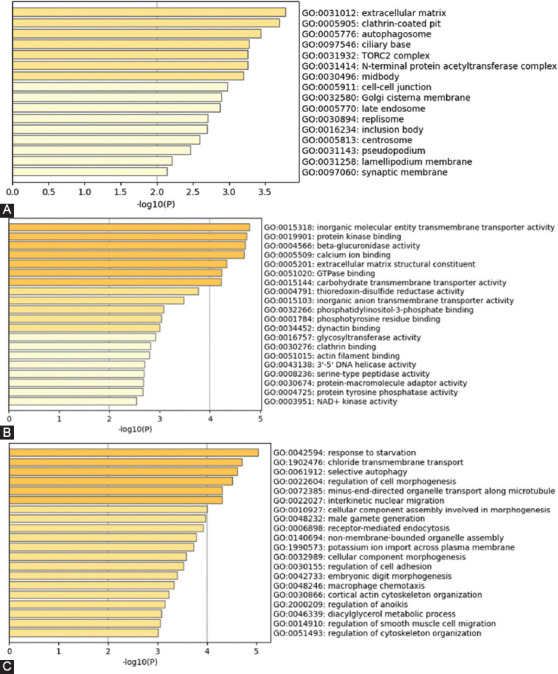
GO function enrichment analysis of candidate genes in OGCT patients. GO enrichment analysis of tumor-specific genes common to 11 patients was retrieved using Metascape software. Significantly (*P*<0.05) enriched GO terms in cellular components (A), molecular functions (B), and biological processes (C) branches are presented. Bar chart of enriched GO term clusters; color intensity statistical significance. OGCT: Ovarian granulosa cell tumor.

**FIGURE 4:**
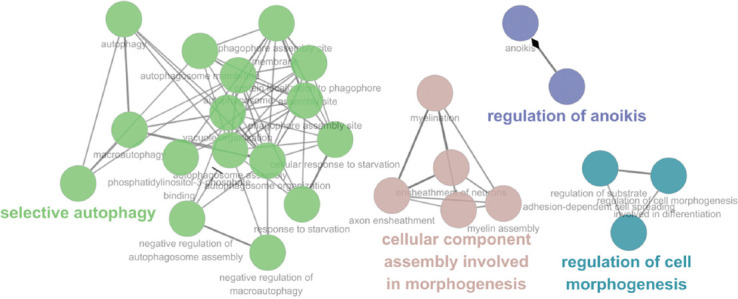
Gene ontology (GO) functional network analysis. Statistically significant enriched GO terms visualized by Cytoscape software add-on ClueGO plugin. *p*-value was adjusted by Benjamini-Hochberg false discovery rate. Node represents each statistically significant enriched GO term. The different size of nodes indicates the number of mapped genes in each GO term. In GO functional network analysis, the most significant and representative GO term is highlighy in bold and the offspring categories are connected with gray title.

To understand the functional relevance, we analyzed the GO of 507 genes (16 overlapping genes in SNPs and indels), including 417 and 106 genes in tumor-specific SNPs and tumor-specific indels, respectively, in CCs, MFs, and BPs through metascape analysis (Figure 3; Table S2). The most significantly enriched gene set in the cellular component is “extracellular matrix (23 genes),” “clathrin-coated pit (16 genes),” “autophagosome (8 genes),” and “ciliary base (10 genes)” (Figure 3A). For the MF, “inorganic molecular entity transmembrane transporter activity (31 genes)” and “protein kinase binding (29 genes)” showed the most significantly enriched (Figure 3B). The most significant gene set enrichment is “response to starvation (20 genes),” “chloride transmembrane transport (21 genes),” “selective autophagy (31 genes),” and “regulation of cell morphogenesis (43 genes)” in the BP (Figure 3C). Interestingly, two candidate genes (*SEC22B* and *FEZ2*), which were highly associated with ovarian cancer in three cancer databases, were identified in the term “selective autophagy”. In addition, we performed gene-gene interaction analysis using ClueGO (*p* < 0.05; BH). The genes were highly enriched in “selective autophagy (9 genes),” “cellular component assembly in morphogenesis (9 genes),” “regulation of anoikis (4 genes),” and “regulation of cell morphogenesis (17 genes)” (Figure 4; Table S3).

### Pathogenic variants in OGCT

To identify pathogenic variants in OGCT, we collected variants with MAF <0.05 using the 1,000 genomes project database, NHLBI exome sequencing project, and ExAC database (http://exac.broadinstitute.org). Of the 16 variants in nine genes found in three cancer databases, we identified seven nonsynonymous SNPs, except unknown variants with MAF ≤ 0.05, including five SNPs (p.Thr343Ile, p.Met357Ile, p.Glu364Ala, p.Glu364Asp, and p.Ser366Thr) in *MUC3A* and two SNPs (p.Ser7Asn and p.Gly8Val) in *PRSS3* (Table 4). In investigating rare functional variants using the public database, Allele Frequency Aggregator, we confirmed that the seven selected variants have an infrequent MAF of 0.0092 on average in the Asian population (Table 4) [33].

**TABLE 4:**
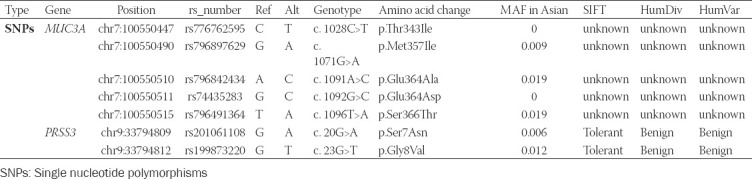
Detail of rare variants with deleterious effects

*PRSS3* is a member of the trypsin family of serine proteases. The serine proteases are secreted by several enzymes that promote tumor growth and metastatic progression in various cancers, including lung adenocarcinoma, prostate cancer, and pancreatic cancer [34,35]. Interestingly, the expression of *PRSS3* showed a significant increase in epithelial ovarian cancer tissue compared to normal ovarian samples at mRNA and protein levels [36]. *MUC3A* is a member of the membrane mucin gene family that encodes secreted and membrane bounding epithelial glycoproteins and also referred to as a potent modifier of epidermal growth factor receptor and is known to lead to poor prognosis by upregulated and downregulated expression of programmed cell death-ligand 1 in non-small cell lung cancer [37]. In addition, we used SIFT and polyphen-2 program to analyze seven variants for the potentially deleterious effects. Of these, two SNPs (p.Ser7Asn and p.Gly8Val) in *PRSS3* were predicted to be tolerant or benign in the SIFT and Polyphen-2 (Table 4). 5 SNP variants in *MUC3A* were predicted to be “unknown” in SIFT and Polyphen-2 (Table 4).

## DISCUSSION

Today, high throughput next-generation sequencing (NGS) is a technology that helps make genetic testing faster and cheaper. WES, one of the NGS technologies, is widely used to investigate the genetic variations and mechanisms of rare diseases and cancers. Although exons account for only about 2% of the human genome, exons contain approximately 85% of the mutations in Mendelian disorders with significant effects [38,39].

In ovarian cancer, there are various factors to increase the risks, such as aging, obesity, hormone therapy after menopause, and smoking. However, the development and causes of OGCT is still unclear. OGCT accounts for about 5% of ovarian cancers, but the prognosis is poor due to a high recurrence rate of over 50%. Thus, early diagnosis and treatment using genetic mutations are important.

Several studies tried to understand the mechanism of occurrence for OGCTs using NGS sequencing. *FOXL2* mutation (C134W) was found using whole-transcriptome paired-end RNA sequencing and whole-genome sequencing [13,40]. The *FOXL2* is strongly expressed in granulosa cells as one of the earliest markers of ovarian differentiation. *FOXL2* C134W mutation is the loss of function mutation that is prevalent in adult OCCT patients. Two *TERT* promoter mutations (C228T and C250T) might be a biomarker of OGCT using WES and targeted sequencing [15]. These two mutations are involved in telomerase activation in several cancers, including central nervous system tumors, hepatocellular carcinomas, bladder cancers, and thyroid cancers. Significantly, they are hot-spot mutations found in about 15.9% of ovarian clear cell carcinomas.

Mucins protect epithelial tissues against external environments under normal physiological conditions [41]. The mucins are a family of O-glycoproteins that play an important role in epithelial cell regeneration, cell adhesions, immune response, and cell signaling. Reduced expression levels of several mucin genes, including *MUC3*, *MUC4*, and *MUC5B* in patients with Crohn’s disease, suggest primary or early mucosal defect of these genes [42]. Chauhan et al. showed that *MUC13* is more overexpressed in malignant ovarian tumors than in benign ovarian tumors [43]. *MUC16* is overexpressed in epithelial ovarian cancer and used as a biomarker (CA125) [44-46]. On the other hand, the expression of *MUC3* and *MUC4* was significantly reduced as the cancer stage increased [47]. *MUC3A* plays a role in the pathogenesis and progression of cancers [48]. Abnormal overexpression of *MUC3A* in clear-cell renal cell carcinoma (ccRcc), breast, pancreatic, gastric, colorectal, appendiceal, and prostate cancer is associated with poor prognosis [49-52]. The abnormal expression of *MUC3A* is highly associated with a poor prognosis in many tumor types, although the roles of *MUC3A* in cancer development are not yet clear. In addition, hypomethylation contributes to the expression of *MUC3A* in cancer cells [53]. The methylation status of *MUC3A* is also utilized as an epigenetic diagnostic marker for carcinogenic risk and prognosis in cancer patients.

Gene enrichment analysis showed that tumor-specific variants are highly enriched in anoikis and autophagy pathways. Anoikis resistance represents a critical and distinguishing feature underlying the aggressiveness of ovarian cancer cells. Several studies reported that enhanced anoikis resistance is closely related to activating the Src/Akt/Erk signaling pathway, which is a critical cellular process including aggressiveness and tumorigenicity [54,55]. In addition, autophagy has been implicated in both tumor suppression and growth, and regulates oncogenic protein substrates and angiogenesis [56,57]. Autophagy can inhibit cancer by preventing angiogenesis in prostate, breast, and colon cancer cells. Cai et al. reported that a high rate of metabolism and autophagy is associated with increased anoikis resistance, and blocking these metabolic pathways significantly increases anoikis and inhibits tumor development *in vitro* and *in vivo* [54].

## CONCLUSION

In summary, we identified five rare variants for the potentially deleterious effects in *MUC3A* though WES. Our findings suggest that *MUC3A* may contribute to OGCT development, although little is known about the functional role of *MUC3A* in cancer pathology. It also suggests that *MUC3A* may be used as a potential biomarker for OGCT. For this, further investigation with more tumor samples is required to understand the development of OGCT.

## SUPPLEMENTARY DATA

Available on: https://www.bjbms.org/ojs/index.php/bjbms/article/view/6789/2433

## References

[1] Rokitansky C (1859). Uber abnormalitiaten des corpus luteum. Allg Wien Med.

[2] Chen YC, Chang LC, Soong RS (2012). A late recurring and easily forgotten tumor:Ovarian granulosa cell tumor. World J Surg Oncol.

[3] Hillman RT, Celestino J, Terranova C, Beird HC, Gumbs C, Little L (2018). KMT2D/MLL2 inactivation is associated with recurrence in adult-type granulosa cell tumors of the ovary. Nat Commun.

[4] Pectasides D, Pectasides E, Psyrri A (2008). Granulosa cell tumor of the ovary. Cancer Treat Rev.

[5] Ukah CO, Ikpeze OC, Eleje GU, Eke AC (2011). Adult granulosa cell tumor associated with endometrial carcinoma:A case report. J Med Case Rep.

[6] Schwartz PE, Smith JP (1976). Treatment of ovarian stromal tumors. Am J Obstet Gynecol.

[7] Ayhan A, Salman MC, Velipasaoglu M, Sakinci M, Yuce K (2009). Prognostic factors in adult granulosa cell tumors of the ovary:A retrospective analysis of 80 cases. J Gynecol Oncol.

[8] Roze J, Monroe G, Kutzera J, Groeneweg J, Stelloo E, Paijens S (2020). Whole genome analysis of ovarian granulosa cell tumors reveals tumor heterogeneity and a high-grade TP53-specific subgroup. Cancers (Basel).

[9] Sun HD, Lin H, Jao MS, Wang KL, Liou WS, Hung YC (2012). A long-term follow-up study of 176 cases with adult-type ovarian granulosa cell tumors. Gynecol Oncol.

[10] Schumer ST, Cannistra SA (2003). Granulosa cell tumor of the ovary. J Clin Oncol.

[11] Jamieson S, Butzow R, Andersson N, Alexiadis M, Unkila-Kallio L, Heikinheimo M (2010). The FOXL2 C134W mutation is characteristic of adult granulosa cell tumors of the ovary. Mod Pathol.

[12] Schrader KA, Gorbatcheva B, Senz J, Heravi-Moussavi A, Melnyk N, Salamanca C (2009). The specificity of the FOXL2 c.402C>G somatic mutation:A survey of solid tumors. PLoS One.

[13] Shah SP, Kobel M, Senz J, Morin RD, Clarke BA, Wiegand KC (2009). Mutation of FOXL2 in granulosa-cell tumors of the ovary. N Engl J Med.

[14] Georges A, L'Hote D, Todeschini AL, Auguste A, Legois B, Zider A (2014). The transcription factor FOXL2 mobilizes estrogen signaling to maintain the identity of ovarian granulosa cells. Elife.

[15] Alexiadis M, Rowley SM, Chu S, Leung DT, Stewart CJ, Amarasinghe KC (2019). Mutational landscape of ovarian adult granulosa cell tumors from whole exome and targeted TERT promoter sequencing. Mol Cancer Res.

[16] Lee HW, Park TI, Jang SY, Park SY, Park WJ, Jung SJ (2017). Clinicopathological characteristics of TERT promoter mutation and telomere length in hepatocellular carcinoma. Medicine (Baltimore).

[17] Zhang Y, Chen Y, Yang C, Seger N, Hesla AC, Tsagkozis P (2021). TERT promoter mutation is an objective clinical marker for disease progression in chondrosarcoma. Mod Pathol.

[18] Mosrati MA, Malmstrom A, Lysiak M, Krysztofiak A, Hallbeck M, Milos P (2015). TERT promoter mutations and polymorphisms as prognostic factors in primary glioblastoma. Oncotarget.

[19] Hamdi Y, Boujemaa M, Ben Rekaya M, Ben Hamda C, Mighri N, El Benna H (2018). Family specific genetic predisposition to breast cancer:Results from Tunisian whole exome sequenced breast cancer cases. J Transl Med.

[20] Kim YC, Soliman AS, Cui J, Ramadan M, Hablas A, Abouelhoda M (2017). Unique features of germline variation in five egyptian familial breast cancer families revealed by exome sequencing. PLoS One.

[21] Chen J, Li Y, Wu J, Liu Y, Kang S (2021). Whole-exome sequencing reveals potential germline and somatic mutations in 60 malignant ovarian germ cell tumorsdagger. Biol Reprod.

[22] Cassandri M, Smirnov A, Novelli F, Pitolli C, Agostini M, Malewicz M (2017). Zinc-finger proteins in health and disease. Cell Death Discov.

[23] Aslan B, Monroig P, Hsu MC, Pena GA, Rodriguez-Aguayo C, Gonzalez-Villasana V (2015). The ZNF304-integrin axis protects against anoikis in cancer. Nat Commun.

[24] Zhang X, Jing Y, Qin Y, Hunsucker S, Meng H, Sui J (2012). The zinc finger transcription factor ZKSCAN3 promotes prostate cancer cell migration. Int J Biochem Cell Biol.

[25] Yang L, Hamilton SR, Sood A, Kuwai T, Ellis L, Sanguino A (2008). The previously undescribed ZKSCAN3 (ZNF306) is a novel “driver”of colorectal cancer progression. Cancer Res.

[26] Li J, Hao N, Han J, Zhang M, Li X, Yang N (2020). ZKSCAN3 drives tumor metastasis via integrin beta4/FAK/AKT mediated epithelial-mesenchymal transition in hepatocellular carcinoma. Cancer Cell Int.

[27] Califano D, Pignata S, Pisano C, Greggi S, Laurelli G, Losito NS (2010). FEZ1/LZTS1 protein expression in ovarian cancer. J Cell Physiol.

[28] Kruse AJ, Croce S, Kruitwagen RF, Riedl RG, Slangen BF, van Gorp T (2014). Aggressive behavior and poor prognosis of endometrial stromal sarcomas with YWHAE-FAM22 rearrangement indicate the clinical importance to recognize this subset. Int J Gynecol Cancer.

[29] Sun W, Tian BX, Wang SH, Liu PJ, Wang YC (2020). The function of SEC22B and its role in human diseases. Cytoskeleton (Hoboken).

[30] Veeraraghavan J, Ma J, Hu Y, Wang XS (2016). Recurrent and pathological gene fusions in breast cancer:Current advances in genomic discovery and clinical implications. Breast Cancer Res Treat.

[31] Tashkandi H, Petrova-Drus K, Batlevi CL, Arcila ME, Roshal M, Sen F (2019). Divergent clonal evolution of a common precursor to mantle cell lymphoma andclassic Hodgkin lymphoma. Cold Spring Harb Mol Case Stud.

[32] Robinson DR, Kalyana-Sundaram S, Wu YM, Shankar S, Cao X, Ateeq B (2011). Functionally recurrent rearrangements of the MAST kinase and Notch gene families in breast cancer. Nat Med.

[33] (2021). ALFA:Allele Frequency Aggregator.

[34] Ma H, Hockla A, Mehner C, Coban M, Papo N, Radisky DC (2019). PRSS3/Mesotrypsin and kallikrein-related peptidase 5 are associated with poor prognosis and contribute to tumor cell invasion and growth in lung adenocarcinoma. Sci Rep.

[35] Jiang G, Cao F, Ren G, Gao D, Bhakta V, Zhang Y (2010). PRSS3 promotes tumour growth and metastasis of human pancreatic cancer. Gut.

[36] Ma R, Ye X, Cheng H, Ma Y, Cui H, Chang X (2015). PRSS3 expression is associated with tumor progression and poor prognosis in epithelial ovarian cancer. Gynecol Oncol.

[37] Luo Y, Ma S, Sun Y, Peng S, Zeng Z, Han L (2021). MUC3A induces PD-L1 and reduces tyrosine kinase inhibitors effects in EGFR-mutant non-small cell lung cancer. Int J Biol Sci.

[38] Rabbani B, Tekin M, Mahdieh N (2014). The promise of whole-exome sequencing in medical genetics. J Hum Genet.

[39] Choi M, Scholl UI, Ji W, Liu T, Tikhonova IR, Zumbo P (2009). Genetic diagnosis by whole exome capture and massively parallel DNA sequencing. Proc Natl Acad Sci USA.

[40] Pilsworth JA, Cochrane DR, Neilson SJ, Moussavi BH, Lai D, Munzur AD (2021). Adult-type granulosa cell tumor of the ovary:A FOXL2-centric disease. J Pathol Clin Res.

[41] Gendler SJ, Spicer AP (1995). Epithelial mucin genes. Annu Rev Physiol.

[42] Buisine MP, Desreumaux P, Debailleul V, Gambiez L, Geboes K, Ectors N (1999). Abnormalities in mucin gene expression in Crohn's disease. Inflamm Bowel Dis.

[43] Chauhan SC, Kumar D, Jaggi M (2009). Mucins in ovarian cancer diagnosis and therapy. J Ovarian Res.

[44] Yin BW, Lloyd KO (2001). Molecular cloning of the CA125 ovarian cancer antigen:Identification as a new mucin, MUC16. J Biol Chem.

[45] Theriault C, Pinard M, Comamala M, Migneault M, Beaudin J, Matte I (2011). MUC16 (CA125) regulates epithelial ovarian cancer cell growth, tumorigenesis and metastasis. Gynecol Oncol.

[46] Felder M, Kapur A, Gonzalez-Bosquet J, Horibata S, Heintz J, Albrecht R (2014). MUC16 (CA125):Tumor biomarker to cancer therapy, a work in progress. Mol Cancer.

[47] Giuntoli RL, Rodriguez GC, Whitaker RS, Dodge R, Voynow JA (1998). Mucin gene expression in ovarian cancers. Cancer Res.

[48] Niu T, Liu Y, Zhang Y, Fu Q, Liu Z, Wang Z (2016). Increased expression of MUC3A is associated with poor prognosis in localized clear-cell renal cell carcinoma. Oncotarget.

[49] Wang RQ, Fang DC (2003). Alterations of MUC1 and MUC3 expression in gastric carcinoma:Relevance to patient clinicopathological features. J Clin Pathol.

[50] Rakha EA, Boyce RW, Abd El-Rehim D, Kurien T, Green AR, Paish EC (2005). Expression of mucins (MUC1, MUC2, MUC3, MUC4, MUC5AC and MUC6) and their prognostic significance in human breast cancer. Mod Pathol.

[51] Park HU, Kim JW, Kim GE, Bae HI, Crawley SC, Yang SC (2003). Aberrant expression of MUC3 and MUC4 membrane-associated mucins and sialyl Le(x) antigen in pancreatic intraepithelial neoplasia. Pancreas.

[52] Leroy X, Devisme L, Buisine MP, Copin MC, Aubert S, Gosselin B (2003). Expression of human mucin genes during normal and abnormal renal development. Am J Clin Pathol.

[53] Yamada N, Kitamoto S, Yokoyama S, Hamada T, Goto M, Tsutsumida H (2011). Epigenetic regulation of mucin genes in human cancers. Clin Epigenetics.

[54] Cai Q, Yan L, Xu Y (2015). Anoikis resistance is a critical feature of highly aggressive ovarian cancer cells. Oncogene.

[55] Kim YN, Koo KH, Sung JY, Yun UJ, Kim H (2012). Anoikis resistance:An essential prerequisite for tumor metastasis. Int J Cell Biol.

[56] White E (2012). Deconvoluting the context-dependent role for autophagy in cancer. Nat Rev Cancer.

[57] Amaravadi R, Kimmelman AC, White E (2016). Recent insights into the function of autophagy in cancer. Genes Dev.

